# Crystal structures of chlorido­[dihy­droxybis­(1-imino­eth­oxy)]arsanido-κ^3^ *N*,*As*,*N*′]platinum(II) and of a polymorph of chlorido­[dihy­droxybis­(1-imino­prop­oxy)arsanido-κ^3^ *N*,*As*,*N*′]platinum(II)

**DOI:** 10.1107/S2056989019016463

**Published:** 2020-01-10

**Authors:** Nina R. Marogoa, D.V. Kama, Hendrik G. Visser, M. Schutte-Smith

**Affiliations:** a University of the Free State, Department of Chemistry, PO Box 339, Nelson Mandela Drive, Bloemfontein, 9301, South Africa

**Keywords:** crystal structure, arsenoplatin, platinum, cisplatin

## Abstract

Each central platinum(II) atom in the crystal structures of chlorido­[dihy­droxybis­(1-imino­eth­oxy)arsanido-κ^3^ *N*,*As*,*N*′]platinum(II), [Pt(C_4_H_10_AsN_2_O_4_)Cl] (**1**), and of chlorido­[dihy­droxybis­(1-imino­prop­oxy)arsanido-κ^3^ *N*,*As*,*N*′]platinum(II), [Pt(C_6_H_14_AsN_2_O_4_)Cl] (**2**), is coordinated by two nitro­gen donor atoms, a chlorido ligand and to arsenic, which, in turn, is coordinated by two oxygen donor ligands, two hydroxyl ligands and the platinum(II) atom. The square-planar and trigonal–bipyramidal coordination environments around platinum and arsenic, respectively, are significantly distorted with the largest outliers being 173.90 (13) and 106.98 (14)° for platinum and arsenic in (**1**), and 173.20 (14)° and 94.20 (9)° for (**2**), respectively. One intra­molecular and four classical inter­molecular hydrogen-bonding inter­actions are observed in the crystal structure of (**1**), which give rise to an infinite three-dimensional network. A similar situation (one intra­molecular and four classical inter­molecular hydrogen-bonding inter­actions) is observed in the crystal structure of (**2**). Various π-inter­actions are present in (**1**) between the platinum(II) atom and the centroid of one of the five-membered rings formed by Pt, As, C, N, O with a distance of 3.7225 (7) Å, and between the centroids of five-membered (Pt, As, C, N, O) rings of neighbouring mol­ecules with distances of 3.7456 (4) and 3.7960 (6) Å. Likewise, weak π-inter­actions are observed in (**2**) between the platinum(II) atom and the centroid of one of the five-membered rings formed by Pt, As, C, N, O with a distance of 3.8213 (2) Å, as well as between the Cl atom and the centroid of a symmetry-related five-membered ring with a distance of 3.8252 (12) Å. Differences between (**2**) and the reported polymorph [Miodragović *et al.* (2013 ▸). *Angew. Chem. Int. Ed.* **52**, 10749–10752] are discussed.

## Abstract 2

The square-planar and trigonal–bipyramidal coordination environment around the Pt and As atom, respectively, each are distorted, with a τ_5_ value of 0.794 and 0.711 for arsenic in (**1**) and (**2**), respectively.

## Chemical context   

Platinum and arsenic compounds have shown great versatility in terms of applications in the biological and medicinal fields (Reedijk, 2009 ▸). Platinum compounds are still the most widely used drugs in the fight against cancer in spite of the serious side effects and the resistance of some types of cancers (Miller *et al.*, 2002 ▸; Basu & Krishnamurthy, 2010 ▸; Jakupec *et al.*, 2003 ▸; Kauffman *et al.*, 2010 ▸; Wheate *et al.*, 2010 ▸; Rosenberg *et al.*, 1965 ▸; Marino *et al.*, 2017 ▸; Aabo *et al.*, 1998 ▸; Kelland, 2007 ▸; Shi *et al.*, 2019 ▸). Tumoral malignancies have a high lethality rate and are among the most widespread and difficult diseases to treat. The need for the development of new drugs and treatment alternatives has increased as many of the available effective drugs are comparable and similar to each other (Ott, 2009 ▸; Burchenal, 1978 ▸). Platinum-based anti­tumour agents have guided and constructed the current tumor chemotherapy treatment, but the side effects complicate and inhibit their clinical application (Rosenberg *et al.*, 1965 ▸; Marino *et al.*, 2017 ▸; Basu & Krishnamurthy, 2010 ▸; Aabo *et al.*, 1998 ▸; Kelland, 2007 ▸; Shi *et al.*, 2019 ▸). Drug resistance is a major limiting factor in terms of the range of tumours that can be treated and the improvement of the therapy (Marino *et al.*, 2017 ▸). Arsenic trioxide was approved by the FDA in 2000 for the treatment of acute promyelocytic leukemia, and since then several studies have shown that the combinatorial employment of arsenic and platinum-based cancer drugs has shown significant therapeutic potential (Wang *et al.*, 2004 ▸; Shen *et al.*, 2004 ▸; Emadi & Gore, 2010 ▸; Zhang *et al.*, 2009 ▸, 2010 ▸). These results led to the synthesis of complexes containing both platinum and arsenic (Swindell *et al.*, 2013 ▸; Miodragović *et al.*, 2013 ▸, 2019 ▸), which were called arsenoplatins. Initial results indicate that these complexes are able to bypass drug-resistance mechanisms that lower the effect of cisplatin and have higher cytotoxicity than cisplatin in some cases. To date, the studies of Miodragović *et al.* (2013 ▸, 2019 ▸) are the only crystallographic data available in the CCDC (Groom *et al.*, 2016 ▸).
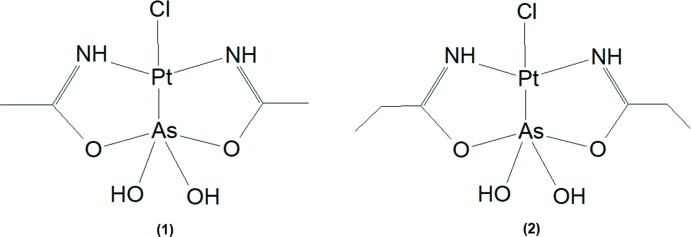


The structures reported here, [Pt(C_4_H_10_AsN_2_O_4_)Cl] (**1**), and [Pt(C_6_H_14_AsN_2_O_4_)Cl], (**2**), expand on this work and form part of an ongoing study on arsenoplatins, their solid- and solution-state behaviour and evaluation thereof.

## Database survey   

Two crystal structures similar to (**1**) were found after a search of the Cambridge Structure Database (CSD, Version 5.40, update of November 2019; Groom *et al.*, 2016 ▸), both of which (ODOHAS, ODOHEW) were reported by Miodragović *et al.* (2013 ▸, 2019 ▸). They consist of the same arsenoplatin complex as (**1**), accompanied by an acetamide hemihydrate and acetamide solvent species in the unit cell, and crystallize in the *P* 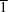 and *P*2_1_/*n* space groups, respectively. The search also revealed that (**2**) represents a polymorph, with the first crystal structure determination (ODOGOF; Miodragović *et al.*, 2013 ▸) in the ortho­rhom­bic space group type *Pbca*, in contrast to space group type *P*2_1_/*c* of (**2**).

## Structural commentary   

**Figure 1:**
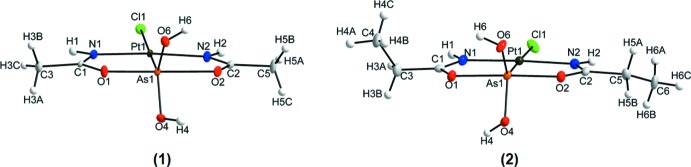
Mol­ecular structures of (**1**) and (**2**), indicating the numbering schemes. Displacement ellipsoids are drawn at a probability level of 50%.

In (**1**) the square-planar coordination environment around platinum(II) is defined by two nitro­gen donor atoms, a chlorido ligand and the coordination to arsenic. In turn, arsenic is coordinated by two oxygen donor atoms, two hydroxyl ligands and by platinum(II), completing a trigonal–bipyramidal coordination sphere (Fig. 1 ▸). The first (ODOHAS) of the other two structure reports with a chlorido­[dihy­droxybis­(1-imino­eth­oxy)]arsanido]platinum(II) mol­ecule (Miodragović *et al.*, 2013 ▸) is different from (**1**) because of an acetamide solvent mol­ecule in the unit cell and a different space group (*P*2_1_/*n*), and the second (ODOHEW) crystallizes in the same space group as (**1**) (*P* 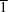) but with acetamide and hemihydrate solvent mol­ecules in the unit cell. The bond lengths in the title compound compare very well with those in the two structures in literature. The Pt—As bond length of 2.2730 (12) Å and the Pt—Cl bond length of 2.3401 (15) Å are similar to 2.2732 (3) and 2.3272 (8) Å for ODOHEW, and 2.2729 (2) and 2.3328 (6) Å for ODOHAS. The Pt—N bond lengths vary between 1.999 (4) and 2.005 (4) Å, the As—O bond lengths between 1.898 (3) and 2.107 (3) Å, and the As—OH bond lengths between 1.722 (3) and 1.738 (3) Å. Overall, these mol­ecular structures compare well. When comparing the Pt—As and Pt—Cl bond lengths to those of other platinum(II) complexes where As and Cl are in *trans* positions, it is clear that the Pt—As bond lengths do not vary significantly and range between 2.3333 (6) and 2.3599 (2) Å, while for the Pt—Cl bond lengths a greater variation is seen, in a range from 2.2917 (4) to 2.3927 (5) Å (Reinholdt & Bendix, 2017 ▸; Clegg, 2016 ▸; Dube *et al.*, 2016 ▸; Imoto *et al.*, 2017 ▸; Muessig *et al.*, 2019 ▸). While the Pt—Cl length in (**1**) compares well with these *trans* complexes, the Pt—As bond length is somewhat smaller. The square-planar coordination around the central platinum(II) atom is distorted with N1—Pt1—N2 and N1—Pt1—As1 being 173.90 (13) and 85.18 (11)°, respectively, deviating from the expected 180 and 90°. The trigonal–bipyramidal coordination around the arsenic atom is significantly distorted with O4—As1—O6, O1—As1—O2 and O1—As1—Pt1 being 106.98 (14), 174.04 (11) and 95.35 (10)°, deviating from the ideal 120, 180 and 90°, respectively. Considering arsenic with a coordination number of 5, the index *τ*_5_ parameter can be used to calculate any potential distortion (Addison *et al.*, 1984 ▸). The *τ*_5_ parameter is defined as (*β* – *α*)/60° with *β* the largest and *α* the second largest angle in the coordination sphere and was calculated as 0.794 for (**1**), suggesting a significantly distorted trigonal–bipyramidal shape around arsenic (*τ*_5_ = 0 for an ideal square pyramid and 1 for an ideal trigonal bipyramid).

The coordination environments of the platinum and arsenic atoms in (**2**) are the same as in (**1**), *i.e.* Pt1 is coordinated by a chlorido ligand, two nitro­gen donor atoms and arsenic, that is additionally bonded to two hydroxyl ligands and two oxygen donor atoms (Fig. 1 ▸). The Pt—As and Pt—Cl bond lengths of 2.2672 (8) Å and 2.3387 (11) Å in (**2**) are virtually identical with the bond lengths of 2.2687 (4) Å and 2.3361 (9) Å, respectively, in the ortho­rhom­bic polymorph reported by Miodragović *et al.* (2013 ▸). Again, these Pt—As and Pt—Cl bond lengths fit well into the ranges reported for other structures where As and Cl are in *trans* positions. The square-planar coordination environment around the platinum(II) atom is similarly distorted in the structures of the two polymorphs, with the ideal 180° (N—Pt—N) and 90° (N—Pt—Cl) angles deviating at 173.59 (13) and 94.68 (9)° for the structure determined by Miodragović *et al.* (2013 ▸) and 173.20 (14) (N1—Pt1—N2) and 94.16 (11)° (N1—Pt1—Cl1) for (**2**), respectively. The largest deviation of the trigonal–bipyramidal coordination sphere of the arsenic atom in the polymorphic structures pertains to the Pt—As—OH angle, with reported values of 129.78 (10) and 124.67 (9)° for the ortho­rhom­bic structure (Miodragović *et al.*, 2013 ▸) and of 130.05 (11) and 124.46 (9)° for (**2**). The τ_5_ parameter for (**2**) is calculated as 0.711.

When comparing the mol­ecules of (**1**) and (**2**), it is clear that they do not differ much in terms of bond lengths and angles, with the only structural difference being the alkyl substituent on the ligand, *viz*. in (**1**) an ethyl and in (**2**) a propyl chain. The bond lengths around platinum are all similar (Pt—As, Pt—Cl, Pt—N) as well as the two pairs of As—OH distances. There is a slight variation in the As—O bond lengths, 1.898 (3) and 2.107 (3) Å for (**1**) and 1.946 (3) and 1.979 (3) Å for (**2**). The N1—Pt1—N2 bond angles are similar [173.90 (13)° for (**1**) and 173.20 (14)° for (**2**)] while there are slight differences for the N—Pt1—As1 and N—Pt—Cl1 bond angles: 85.18 (11) and 89.42 (11)° for (**1**), and 87.25 (10) and 86.05 (10)° for (**2**) (N1—Pt1—As1, N2—Pt1—As1), and 93.28 (11) and 92.34 (11)° for (**1**) and 94.16 (11) and 92.53 (10)° for (**2**) (N1—Pt1—Cl1, N2—Pt1—Cl1). The As1—Pt1—Cl1 bond angles also vary being 174.79 (3) and 178.51 (3)° for (**1**) and (**2**). The bond angles around arsenic are all in a similar range but have more variation in some of the angles, for instance 126.42 (10) and 130.05 (11)° (O6—As1—Pt1), 90.24 (9) and 94.20 (9)° (O2—As1—Pt1), and 95.35 (10) and 93.12 (8)° (O1—As1—Pt1) for (**1**) and (**2** *)*, respectively. The trigonal– bipyramidal coord­ination environment around arsenic is distorted in both mol­ecules with a τ_5_ parameter value of 0.794 and 0.711 for (**1**) and (**2**). Thus, the As atom in (**2**) shows a slightly higher distortion than in (**1**).

## Supra­molecular features   

**Figure 2:**
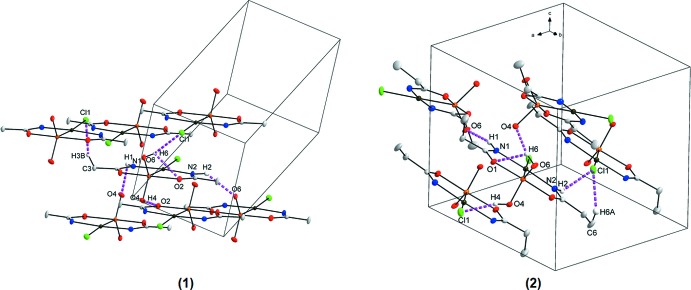
Hydrogen-bonding inter­actions (indicated by purple dashed lines) observed in the structures of (**1**) and (**2**). Hydrogen atoms not involved in hydrogen-bonding inter­actions were omitted for clarity.

**Figure 3:**
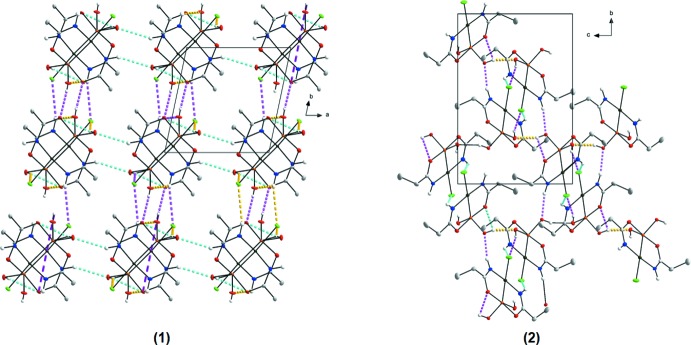
Illustration of the infinite three-dimensional frameworks formed by the hydrogen-bonding inter­actions in (**1**) and (**2**). Blue dashed lines indicate the infinite networks along the *a* axes, purple dashed lines along the *b* axes and gold dashed lines along the *c* axes. Hydrogen atoms not involved in the inter­actions were omitted for clarity.

**Figure 4:**
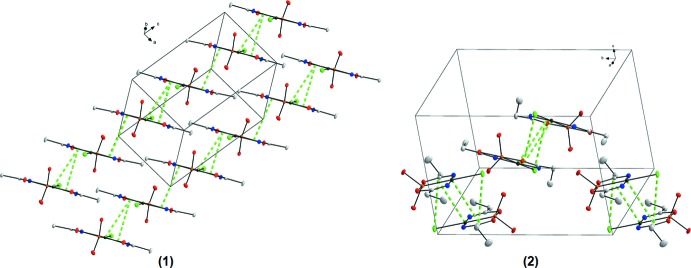
π-inter­actions observed in the crystal structures of (**1**) and (**2**). Hydrogen atoms were omitted for clarity.

In the crystal structure of (**1**), six hydrogen-bonding inter­actions are observed (Table 1 ▸), five inter­molecular (N1—H1⋯O4^i^, N2—H2⋯O6^ii^, O4—H4⋯O2^iii^, O6—H6⋯Cl1^iv^, C3—H3*B*⋯Cl^v^) and one intra­molecular (O6—H6⋯O2), as illustrated in Fig. 2 ▸. Bifurcation creates inter- and intra­molecular inter­actions that can contribute to the stability of the structure. One of the donor hydrogen atoms (H6) takes part in hydrogen-bonding inter­actions to an oxygen atom (O2) and a chloride atom (Cl1) and forms an unsymmetrical bifurcated bond. Overall, the four stronger inter­molecular hydrogen-bonding inter­actions sustain an infinite three-dimensional framework (Fig. 3 ▸). An inter­molecular cluster is formed from the strongest hydrogen-bonding inter­action [O4⋯O2^iii^ = 2.750 (4) Å], which generates an infinite chain along the *c-*axis direction (as can be seen in Fig. 3 ▸). Various π-inter­actions are also observed in (**1**), defined by the platinum(II) atom of one mol­ecule to the centroid of the (Pt1,As1,C2,N2,O2) ring with a Pt⋯centroid distance of 3.7225 (7) Å, by the centroid of the (Pt1,As1,C2,N2,O2) ring to the centroid of the (Pt1,As1,C2,N2,O2) (−*x*, −*y*, 1 − *z*) ring of an adjacent mol­ecule with a distance of 3.7456 (4) Å, and by the centroid of the (Pt1,As1,C1,N1,O1) ring to the centroid of another (Pt1,As1,C1,N1,O2) (−*x*, −*y*, −*z*) ring with a distance of 3.7960 (6) Å (Fig. 4 ▸). When viewed along the *c* axis, individual mol­ecules pack in ‘column-like’ structures in an alternating head-to-tail fashion, as illustrated in Fig. 3 ▸.

The crystal structure of (**2**) is likewise stabilized by one intra­molecular (O6—H6⋯O1) and five inter­molecular (N1—H1⋯O6^i^, N2—H2⋯Cl1^ii^, O4—H4⋯Cl1^iii^, O6—H6⋯O4^iv^, C6—H6*A*⋯Cl1^ii^) hydrogen-bonding inter­actions (Table 2 ▸, Fig. 2 ▸), again with an unsymmetrical bifurcated hydrogen bond involving atom H6 (bonding to O1 and O4) and a resulting three-dimensional network structure, as illustrated in Fig. 3 ▸. In addition, Cl1 is the acceptor of two hydrogen-bonding inter­actions. Two weak π-inter­actions are also observed in (**2**), one from Pt1 to the centroid of Pt1,As1,O1,C1,N1 with a distance of 3.8213 (2) Å and the other from Cl1 to a symmetry-related centroid (Pt1,As1,O1,C1,N1; 1 − *x*, −*y*, −*z*) with a distance of 3.8252 (12) Å (Fig. 4 ▸).

In comparison, mol­ecules in (**1**) and (**2**) pack differently due to the presence of different alkyl chains (Fig. 3 ▸).

## Synthesis and crystallization   

**Synthesis of 1**

K_2_PtCl_4_ (416 mg, 1 mmol) was added to a 125 ml solution of 9:1 (*v*:*v*) CH_3_CN/H_2_O. The mixture was stirred at 363 K. Once the K_2_PtCl_4_ had dissolved, As_2_O_3_ (405 mg, 2.05 mmol) was added to the solution and refluxed at 363 K for 48 h. The mixture was then filtered, and the filtrate was left to stand at room temperature. Crystals suitable for X-ray crystallography were obtained by slow evaporation. Yield: 301 mg (66%). ^1^H NMR (300.18 MHz, dimethyl sulfoxide-*d*_6_): σ = 7.30 (OH, 2H,*s*), 6.69 (NH, 2H,*s*), 1.75 (CH_3_, 6H, *s*) ppm. ^13^C NMR (150.95 MHz, dimethyl sulfoxide-*d*_6_): σ =172 (CN), 23 (CH_3_) ppm. ^195^Pt NMR (242.99 MHz, dimethyl sulfoxide-*d*_6_): σ = −3590.62 ppm. UV/Vis = λ = 285 nm, ∊ = 4029 dm^3^ mol^−1^ cm^−1^. Analysis calculated: C, 10.55; H, 2.21; N, 6.15. Found: C, 10.64; H, 2.22; N, 6.09%.

**Synthesis of 2**

K_2_PtCl_4_ (422 mg, 1.02 mmol) was added to a 50 ml solution of 9:1 (*v*:*v*) H_2_O/CH_3_CH_2_CN. The mixture was stirred at room temperature. Once the K_2_PtCl_4_ had dissolved, As_2_O_3_ (400 mg, 2.02 mmol) was added to the solution and was stirred at room temperature for 96 h. The solution was cooled in an ice bath and then filtered. The filtrate was left to stand at room temperature. Crystals suitable for X-ray crystallography were obtained by slow evaporation. Yield: 278 mg (56%). ^1^H NMR (600.28 MHz, dimethyl sulfoxide-*d*_6_): δ = 8.89 (OH, 2H, *s*), 7.99 (NH, 2H, *s*), 2.48 (CH_2_, 4H, *q*), 1.04 (CH_3_, 6H, *t*). ^13^C NMR (150.95 MHz, dimethyl sulfoxide-*d*_6_): δ = 176.18 (CN), 24.53 (CH_2_), 11.70 (CH_3_). ^195^Pt NMR (242.99 MHz, dimethyl sulfoxide-*d*_6_) = −3591.52. UV/Vis: λ_max_ = 270 nm, ∊ = 4231 L mol^−1^ cm^−1^. Analysis calculated C, 14.90; H, 2.92; N, 5.79. Found: C, 14.82; H, 2.91; N, 5.76.

## Refinement   

Crystal data and details of data collections and structure refinements are summarized in Table 3 ▸. Methyl and methyl­ene hydrogen atoms were placed in geometrically idealized positions (C—H = 0.95–0.97 Å) and constrained to ride on their parent atoms [*U*_iso_(H) = 1.5*U*_eq_(C) and 1.2*U*_eq_(C)]. The OH and NH hydrogen atoms were located in a difference-Fourier map and their positional parameters were constrained with O—H = 0.84 (2) Å and N—H = 0.89 (2) Å for (**1**), and N—H = 0.87 (2) Å for (**2**) with O—H distances fixed at 0.82 Å and with *U*_iso_(H) = 1.5*U*_eq_(O). For (**2**), the *F_c_ versus F_o_* plot proved ten reflections to be outliers, and they were removed from the refinement as systematic errors.

## Supplementary Material

Crystal structure: contains datablock(s) global, 1, 2. DOI: 10.1107/S2056989019016463/wm5532sup1.cif

CCDC references: 1938096, 1938095

Additional supporting information:  crystallographic information; 3D view; checkCIF report

## Figures and Tables

**Table 1:** Hydrogen-bond geometry (Å, °) for **1** [Chem scheme1]

*D*—H⋯*A*	*D*—H	H⋯*A*	*D*⋯*A*	*D*—H⋯*A*
N1—H1⋯O4^i^	0.901 (19)	2.50 (5)	3.077 (5)	122 (4)
N2—H2⋯O6^ii^	0.90 (2)	2.51 (4)	3.280 (5)	143 (5)
O4—H4⋯O2^iii^	0.821 (19)	1.93 (2)	2.750 (4)	173 (6)
O6—H6⋯O2	0.827 (19)	2.27 (5)	2.643 (4)	108 (4)
O6—H6⋯Cl1^iv^	0.827 (19)	2.45 (3)	3.191 (3)	149 (5)
C3—H3*B*⋯Cl1^v^	0.96	2.72	3.613 (5)	155

Symmetry codes: (i) 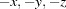; (ii) 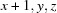; (iii) 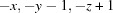; (iv) 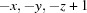; (v) 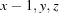.

**Table 2:** Hydrogen-bond geometry (Å, °) for **2** [Chem scheme1]

*D*—H⋯*A*	*D*—H	H⋯*A*	*D*⋯*A*	*D*—H⋯*A*
N1—H1⋯O6^i^	0.88 (5)	2.19 (5)	3.041 (4)	163 (5)
N2—H2⋯Cl1^ii^	0.854 (19)	2.71 (4)	3.408 (4)	140 (4)
O4—H4⋯Cl1^iii^	0.82	2.28	3.071 (3)	161
O6—H6⋯O1	0.82	2.34	2.608 (4)	100
O6—H6⋯O4^iv^	0.82	1.92	2.712 (4)	162
C6—H6*A*⋯Cl1^ii^	0.96	2.82	3.735 (5)	159

Symmetry codes: (i) 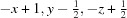; (ii) 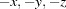; (iii) 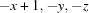; (iv) 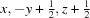.

**Table 3:** Experimental details

	(**1**)	**2**
Crystal data		
Chemical formula	[PtCl(C_4_H_10_AsN_2_O_4_)]	[Pt(C_6_H_14_AsN_2_O_4_)Cl]
*M*_r_	455.59	483.65
Crystal system, space group	Triclinic, *P* 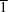	Monoclinic, *P*2_1_/*c*
Temperature (K)	100	100
*a*, *b*, *c* (Å)	7.272 (1), 8.099 (1), 9.350 (2)	8.9009 (3), 14.1270 (5), 9.6438 (3)
α, β, γ (°)	66.588 (5), 83.993 (5), 76.737 (5)	90, 98.243 (2), 90
*V* (Å^3^)	491.81 (14)	1200.11 (9)
*Z*	2	4
Radiation type	Mo *K*α	Mo *K*α
μ (mm^−1^)	17.86	14.65
Crystal size (mm)	0.39 × 0.29 × 0.14	0.55 × 0.42 × 0.08

Data collection		
Diffractometer	Bruker APEXII CCD	Bruker APEXII CCD
Absorption correction	Multi-scan (*SADABS*; Bruker, 2012 ▸)	Multi-scan (*SADABS*; Bruker, 2012 ▸)
*T*_min_, *T*_max_	0.003, 0.090	0.001, 0.301
No. of measured, independent and observed [*I* > 2σ(*I*)] reflections	8305, 2435, 2345	41298, 2891, 2700
*R*_int_	0.039	0.047
(sin θ/λ)_max_ (Å^−1^)	0.667	0.661

Refinement		
*R*[*F* ^2^ > 2σ(*F* ^2^)], *wR*(*F* ^2^), *S*	0.020, 0.052, 1.18	0.022, 0.081, 1.05
No. of reflections	2411	2891
No. of parameters	136	148
No. of restraints	4	1
H-atom treatment	H atoms treated by a mixture of independent and constrained refinement	H atoms treated by a mixture of independent and constrained refinement
Δρ_max_, Δρ_min_ (e Å^−3^)	0.79, −2.05	1.53, −1.98

Computer programs: *APEX2* and *SAINT* (Bruker, 2012 ▸), *SHELXS97* (Sheldrick, 2008 ▸), *SHELXL2014* (Sheldrick, 2015 ▸), *DIAMOND* (Brandenburg & Putz, 2005 ▸) and *WinGX* (Farrugia, 2012 ▸).

## supplementary crystallographic information

### Chlorido[dihydroxybis(1-iminoethoxy)arsanido-κN,As,N']platinum(II) (1) Crystal data

**Table:**

[Pt(C_4_H_10_AsN_2_O_4_)Cl]	*Z* = 2
*M**_r_* = 455.59	*F*(000) = 416
Triclinic, *P*1	*D*_x_ = 3.076 Mg m^−^^3^
Hall symbol: -P 1	Mo *K*α radiation, λ = 0.71069 Å
*a* = 7.272 (1) Å	Cell parameters from 6890 reflections
*b* = 8.099 (1) Å	θ = 3.6–28.3°
*c* = 9.350 (2) Å	µ = 17.86 mm^−^^1^
α = 66.588 (5)°	*T* = 100 K
β = 83.993 (5)°	Cuboid, colourless
γ = 76.737 (5)°	0.39 × 0.29 × 0.14 mm
*V* = 491.81 (14) Å^3^	

### Chlorido[dihydroxybis(1-iminoethoxy)arsanido-κN,As,N']platinum(II) (1) Data collection

**Table:**

Bruker APEXII CCD diffractometer	2345 reflections with *I* > 2σ(*I*)
φ and ω scans	*R*_int_ = 0.039
Absorption correction: multi-scan (SADABS; Bruker, 2012)	θ_max_ = 28.3°, θ_min_ = 3.8°
*T*_min_ = 0.003, *T*_max_ = 0.090	*h* = −9→9
8305 measured reflections	*k* = −8→10
2435 independent reflections	*l* = −12→12

### Chlorido[dihydroxybis(1-iminoethoxy)arsanido-κN,As,N']platinum(II) (1) Refinement

**Table:**

Refinement on *F*^2^	4 restraints
Least-squares matrix: full	Hydrogen site location: mixed
*R*[*F*^2^ > 2σ(*F*^2^)] = 0.020	H atoms treated by a mixture of independent and constrained refinement
*wR*(*F*^2^) = 0.052	*w* = 1/[σ^2^(*F*_o_^2^) + (0.017*P*)^2^ + 1.0064*P*] where *P* = (*F*_o_^2^ + 2*F*_c_^2^)/3
*S* = 1.18	(Δ/σ)_max_ = 0.001
2411 reflections	Δρ_max_ = 0.79 e Å^−^^3^
136 parameters	Δρ_min_ = −2.04 e Å^−^^3^

### Chlorido[dihydroxybis(1-iminoethoxy)arsanido-κN,As,N']platinum(II) (1) Special details

**Table:**

Geometry. All esds (except the esd in the dihedral angle between two l.s. planes) are estimated using the full covariance matrix. The cell esds are taken into account individually in the estimation of esds in distances, angles and torsion angles; correlations between esds in cell parameters are only used when they are defined by crystal symmetry. An approximate (isotropic) treatment of cell esds is used for estimating esds involving l.s. planes.

### Chlorido[dihydroxybis(1-iminoethoxy)arsanido-κN,As,N']platinum(II) (1) Fractional atomic coordinates and isotropic or equivalent isotropic displacement parameters (Å^2^)

**Table:**

	*x*	*y*	*z*	*U*_iso_*/*U*_eq_	
Pt1	0.07024 (2)	0.05975 (2)	0.24814 (2)	0.00525 (6)	
As1	−0.10555 (5)	−0.15921 (5)	0.31382 (4)	0.00631 (9)	
Cl1	0.22859 (14)	0.30050 (13)	0.18937 (12)	0.01233 (19)	
O4	−0.0662 (4)	−0.3392 (4)	0.2529 (3)	0.0115 (6)	
O1	−0.2849 (4)	−0.0205 (4)	0.1539 (3)	0.0113 (6)	
C3	−0.3872 (6)	0.2425 (6)	−0.0760 (5)	0.0130 (8)	
H3B	−0.508229	0.285642	−0.037137	0.019*	
H3A	−0.400172	0.162257	−0.125455	0.019*	
H3C	−0.339248	0.345439	−0.150284	0.019*	
C5	0.3527 (6)	−0.3940 (6)	0.6461 (5)	0.0136 (8)	
H5A	0.451272	−0.333216	0.646809	0.02*	
H5C	0.407126	−0.505519	0.630569	0.02*	
H5B	0.285185	−0.422631	0.743999	0.02*	
O2	0.0724 (4)	−0.3296 (4)	0.5035 (3)	0.0098 (6)	
N2	0.2483 (5)	−0.1101 (5)	0.4204 (4)	0.0098 (7)	
O6	−0.2912 (4)	−0.1890 (4)	0.4486 (3)	0.0109 (6)	
N1	−0.1134 (5)	0.2051 (5)	0.0754 (4)	0.0099 (7)	
C1	−0.2535 (6)	0.1403 (5)	0.0559 (4)	0.0083 (7)	
C2	0.2192 (6)	−0.2701 (5)	0.5165 (5)	0.0090 (7)	
H2	0.355 (5)	−0.091 (8)	0.448 (6)	0.025 (15)*	
H1	−0.113 (8)	0.314 (4)	−0.004 (5)	0.025 (15)*	
H6	−0.236 (7)	−0.202 (7)	0.527 (4)	0.017 (13)*	
H4	−0.059 (8)	−0.438 (4)	0.328 (5)	0.026 (15)*	

### Chlorido[dihydroxybis(1-iminoethoxy)arsanido-κN,As,N']platinum(II) (1) Atomic displacement parameters (Å^2^)

**Table:**

	*U*^11^	*U*^22^	*U*^33^	*U*^12^	*U*^13^	*U*^23^
Pt1	0.00569 (9)	0.00337 (9)	0.00559 (9)	−0.00199 (6)	−0.00017 (6)	−0.00001 (6)
As1	0.00695 (19)	0.00417 (19)	0.00666 (18)	−0.00267 (14)	−0.00050 (14)	0.00001 (14)
Cl1	0.0149 (5)	0.0096 (4)	0.0132 (4)	−0.0071 (4)	0.0013 (4)	−0.0029 (4)
O4	0.0210 (15)	0.0055 (13)	0.0089 (13)	−0.0049 (11)	−0.0006 (11)	−0.0024 (11)
O1	0.0107 (14)	0.0101 (14)	0.0100 (13)	−0.0045 (11)	−0.0032 (11)	0.0013 (11)
C3	0.0101 (19)	0.014 (2)	0.0105 (18)	0.0002 (15)	−0.0032 (15)	−0.0015 (15)
C5	0.0084 (18)	0.0122 (19)	0.0143 (19)	−0.0030 (15)	−0.0055 (15)	0.0029 (15)
O2	0.0107 (14)	0.0059 (13)	0.0098 (13)	−0.0049 (10)	−0.0003 (11)	0.0016 (11)
N2	0.0068 (16)	0.0103 (17)	0.0109 (16)	−0.0039 (13)	−0.0017 (13)	−0.0010 (13)
O6	0.0062 (13)	0.0161 (14)	0.0095 (13)	−0.0038 (11)	0.0006 (11)	−0.0034 (11)
N1	0.0128 (17)	0.0059 (16)	0.0091 (16)	−0.0024 (13)	−0.0009 (13)	−0.0005 (13)
C1	0.0108 (18)	0.0048 (17)	0.0065 (17)	0.0022 (14)	−0.0007 (14)	−0.0013 (14)
C2	0.0082 (18)	0.0074 (18)	0.0092 (17)	0.0002 (14)	0.0004 (14)	−0.0021 (14)

### Chlorido[dihydroxybis(1-iminoethoxy)arsanido-κN,As,N']platinum(II) (1) Geometric parameters (Å, º)

**Table:**

Pt1—N1	1.999 (4)	C3—H3A	0.96
Pt1—N2	2.005 (4)	C3—H3C	0.96
Pt1—As1	2.2730 (12)	C5—C2	1.501 (5)
Pt1—Cl1	2.3401 (15)	C5—H5A	0.96
As1—O4	1.722 (3)	C5—H5C	0.96
As1—O6	1.738 (3)	C5—H5B	0.96
As1—O1	1.898 (3)	O2—C2	1.302 (5)
As1—O2	2.107 (3)	N2—C2	1.301 (5)
O4—H4	0.821 (19)	N2—H2	0.90 (2)
O1—C1	1.316 (5)	O6—H6	0.827 (19)
C3—C1	1.491 (5)	N1—C1	1.306 (5)
C3—H3B	0.96	N1—H1	0.901 (19)
			
N1—Pt1—N2	173.90 (13)	H3B—C3—H3C	109.5
N1—Pt1—As1	85.18 (11)	H3A—C3—H3C	109.5
N2—Pt1—As1	89.42 (11)	C2—C5—H5A	109.5
N1—Pt1—Cl1	93.28 (11)	C2—C5—H5C	109.5
N2—Pt1—Cl1	92.34 (11)	H5A—C5—H5C	109.5
As1—Pt1—Cl1	174.79 (3)	C2—C5—H5B	109.5
O4—As1—O6	106.98 (14)	H5A—C5—H5B	109.5
O4—As1—O1	90.06 (14)	H5C—C5—H5B	109.5
O6—As1—O1	88.71 (14)	C2—O2—As1	116.5 (2)
O4—As1—O2	88.29 (13)	C2—N2—Pt1	122.5 (3)
O6—As1—O2	86.29 (13)	C2—N2—H2	109 (4)
O1—As1—O2	174.04 (11)	Pt1—N2—H2	129 (4)
O4—As1—Pt1	126.37 (10)	As1—O6—H6	100 (4)
O6—As1—Pt1	126.42 (10)	C1—N1—Pt1	121.7 (3)
O1—As1—Pt1	95.35 (10)	C1—N1—H1	109 (4)
O2—As1—Pt1	90.24 (9)	Pt1—N1—H1	130 (4)
As1—O4—H4	111 (4)	N1—C1—O1	121.2 (4)
C1—O1—As1	116.4 (3)	N1—C1—C3	122.7 (4)
C1—C3—H3B	109.5	O1—C1—C3	116.1 (4)
C1—C3—H3A	109.5	N2—C2—O2	121.1 (4)
H3B—C3—H3A	109.5	N2—C2—C5	121.7 (4)
C1—C3—H3C	109.5	O2—C2—C5	117.2 (3)
			
O4—As1—O1—C1	−123.0 (3)	As1—O1—C1—C3	175.8 (3)
O6—As1—O1—C1	130.0 (3)	Pt1—N2—C2—O2	1.2 (6)
Pt1—As1—O1—C1	3.5 (3)	Pt1—N2—C2—C5	−179.1 (3)
Pt1—N1—C1—O1	3.2 (6)	As1—O2—C2—N2	2.8 (5)
Pt1—N1—C1—C3	−177.2 (3)	As1—O2—C2—C5	−177.0 (3)
As1—O1—C1—N1	−4.6 (5)		

### Chlorido[dihydroxybis(1-iminoethoxy)arsanido-κN,As,N']platinum(II) (1) Hydrogen-bond geometry (Å, º)

**Table:**

*D*—H···*A*	*D*—H	H···*A*	*D*···*A*	*D*—H···*A*
N1—H1···O4^i^	0.901 (19)	2.50 (5)	3.077 (5)	122 (4)
N2—H2···O6^ii^	0.90 (2)	2.51 (4)	3.280 (5)	143 (5)
O4—H4···O2^iii^	0.821 (19)	1.93 (2)	2.750 (4)	173 (6)
O6—H6···O2	0.827 (19)	2.27 (5)	2.643 (4)	108 (4)
O6—H6···Cl1^iv^	0.827 (19)	2.45 (3)	3.191 (3)	149 (5)
C3—H3*B*···Cl1^v^	0.96	2.72	3.613 (5)	155

Symmetry codes: (i) −*x*, −*y*, −*z*; (ii) *x*+1, *y*, *z*; (iii) −*x*, −*y*−1, −*z*+1; (iv) −*x*, −*y*, −*z*+1; (v) *x*−1, *y*, *z*.

### Chlorido[dihydroxybis(1-iminopropoxy)arsanido-κN,As,N']platinum(II) (2) Crystal data

**Table:**

[Pt(C_6_H_14_AsN_2_O_4_)Cl]	*F*(000) = 896
*M**_r_* = 483.65	*D*_x_ = 2.677 Mg m^−^^3^
Monoclinic, *P*2_1_/*c*	Mo *K*α radiation, λ = 0.71069 Å
Hall symbol: -P 2ybc	Cell parameters from 9966 reflections
*a* = 8.9009 (3) Å	θ = 3.6–28.4°
*b* = 14.1270 (5) Å	µ = 14.65 mm^−^^1^
*c* = 9.6438 (3) Å	*T* = 100 K
β = 98.243 (2)°	Plate, colourless
*V* = 1200.11 (9) Å^3^	0.55 × 0.42 × 0.08 mm
*Z* = 4	

### Chlorido[dihydroxybis(1-iminopropoxy)arsanido-κN,As,N']platinum(II) (2) Data collection

**Table:**

Bruker APEXII CCD diffractometer	2700 reflections with *I* > 2σ(*I*)
φ and ω scans	*R*_int_ = 0.047
Absorption correction: multi-scan (SADABS; Bruker, 2012)	θ_max_ = 28.0°, θ_min_ = 4.4°
*T*_min_ = 0.001, *T*_max_ = 0.301	*h* = −11→11
41298 measured reflections	*k* = −18→18
2891 independent reflections	*l* = −12→12

### Chlorido[dihydroxybis(1-iminopropoxy)arsanido-κN,As,N']platinum(II) (2) Refinement

**Table:**

Refinement on *F*^2^	Primary atom site location: heavy-atom method
Least-squares matrix: full	Hydrogen site location: mixed
*R*[*F*^2^ > 2σ(*F*^2^)] = 0.022	H atoms treated by a mixture of independent and constrained refinement
*wR*(*F*^2^) = 0.081	*w* = 1/[σ^2^(*F*_o_^2^) + (0.0629*P*)^2^ + 1.201*P*] where *P* = (*F*_o_^2^ + 2*F*_c_^2^)/3
*S* = 1.05	(Δ/σ)_max_ < 0.001
2891 reflections	Δρ_max_ = 1.53 e Å^−^^3^
148 parameters	Δρ_min_ = −1.97 e Å^−^^3^
1 restraint	

### Chlorido[dihydroxybis(1-iminopropoxy)arsanido-κN,As,N']platinum(II) (2) Special details

**Table:**

Geometry. All esds (except the esd in the dihedral angle between two l.s. planes) are estimated using the full covariance matrix. The cell esds are taken into account individually in the estimation of esds in distances, angles and torsion angles; correlations between esds in cell parameters are only used when they are defined by crystal symmetry. An approximate (isotropic) treatment of cell esds is used for estimating esds involving l.s. planes.

### Chlorido[dihydroxybis(1-iminopropoxy)arsanido-κN,As,N']platinum(II) (2) Fractional atomic coordinates and isotropic or equivalent isotropic displacement parameters (Å^2^)

**Table:**

	*x*	*y*	*z*	*U*_iso_*/*U*_eq_	
Pt1	0.33235 (2)	0.06675 (2)	0.08616 (2)	0.00918 (9)	
As1	0.47791 (4)	0.19900 (3)	0.11318 (4)	0.00918 (11)	
Cl1	0.17867 (12)	−0.06796 (6)	0.05304 (12)	0.0183 (2)	
O4	0.6100 (3)	0.2309 (2)	0.0038 (3)	0.0127 (5)	
H4	0.676165	0.190233	0.007601	0.019*	
O1	0.6421 (3)	0.13513 (19)	0.2392 (3)	0.0127 (5)	
O6	0.4810 (3)	0.2897 (2)	0.2331 (3)	0.0150 (6)	
H6	0.524135	0.27179	0.309457	0.022*	
C5	0.1062 (5)	0.2938 (3)	−0.1667 (4)	0.0168 (8)	
H5B	0.167052	0.336047	−0.21515	0.02*	
H5A	0.047602	0.332619	−0.110998	0.02*	
C2	0.2107 (5)	0.2323 (3)	−0.0697 (4)	0.0127 (7)	
N2	0.1856 (4)	0.1445 (2)	−0.0450 (3)	0.0134 (6)	
C4	0.7743 (6)	0.0425 (4)	0.5043 (5)	0.0297 (10)	
H4B	0.79075	0.109321	0.495994	0.045*	
H4A	0.861994	0.014145	0.557876	0.045*	
H4C	0.68723	0.032054	0.550648	0.045*	
O2	0.3329 (3)	0.27611 (19)	−0.0081 (3)	0.0130 (5)	
C1	0.6203 (5)	0.0463 (3)	0.2667 (4)	0.0119 (7)	
C3	0.7473 (5)	−0.0015 (3)	0.3597 (4)	0.0180 (8)	
H3B	0.8395	0.002883	0.317329	0.022*	
H3A	0.723211	−0.068067	0.368096	0.022*	
N1	0.4959 (4)	0.0020 (2)	0.2166 (3)	0.0129 (6)	
C6	−0.0037 (5)	0.2407 (3)	−0.2757 (5)	0.0260 (10)	
H6B	0.052289	0.198648	−0.327478	0.039*	
H6C	−0.058897	0.285263	−0.338787	0.039*	
H6A	−0.073555	0.204823	−0.229549	0.039*	
H1	0.489 (6)	−0.057 (4)	0.244 (6)	0.026 (17)*	
H2	0.106 (4)	0.120 (4)	−0.090 (5)	0.023 (14)*	

### Chlorido[dihydroxybis(1-iminopropoxy)arsanido-κN,As,N']platinum(II) (2) Atomic displacement parameters (Å^2^)

**Table:**

	*U*^11^	*U*^22^	*U*^33^	*U*^12^	*U*^13^	*U*^23^
Pt1	0.01054 (13)	0.00700 (12)	0.00989 (12)	−0.00034 (4)	0.00112 (8)	−0.00025 (4)
As1	0.0127 (2)	0.00657 (19)	0.00790 (18)	−0.00061 (13)	0.00022 (15)	−0.00002 (12)
Cl1	0.0126 (5)	0.0103 (5)	0.0318 (6)	−0.0023 (3)	0.0029 (4)	−0.0018 (3)
O4	0.0132 (14)	0.0138 (12)	0.0109 (11)	−0.0004 (10)	0.0009 (10)	0.0029 (10)
O1	0.0149 (14)	0.0097 (12)	0.0127 (12)	−0.0006 (10)	−0.0008 (10)	0.0028 (10)
O6	0.0258 (16)	0.0084 (13)	0.0092 (12)	0.0020 (11)	−0.0026 (11)	−0.0014 (9)
C5	0.014 (2)	0.0172 (19)	0.0180 (18)	0.0012 (15)	−0.0003 (15)	0.0040 (15)
C2	0.0152 (19)	0.0142 (18)	0.0086 (15)	0.0040 (15)	0.0014 (13)	−0.0010 (13)
N2	0.0125 (16)	0.0132 (16)	0.0143 (15)	−0.0009 (13)	0.0008 (13)	0.0009 (12)
C4	0.032 (3)	0.035 (3)	0.019 (2)	0.010 (2)	−0.0082 (19)	−0.001 (2)
O2	0.0139 (14)	0.0104 (12)	0.0139 (12)	−0.0002 (10)	−0.0010 (10)	0.0018 (10)
C1	0.016 (2)	0.0103 (16)	0.0107 (16)	0.0016 (15)	0.0053 (14)	−0.0001 (13)
C3	0.020 (2)	0.0132 (18)	0.0191 (18)	0.0038 (15)	−0.0020 (16)	0.0029 (14)
N1	0.0178 (17)	0.0085 (15)	0.0122 (14)	0.0028 (12)	0.0013 (12)	0.0019 (12)
C6	0.023 (2)	0.029 (2)	0.022 (2)	0.0019 (18)	−0.0081 (18)	0.0108 (18)

### Chlorido[dihydroxybis(1-iminopropoxy)arsanido-κN,As,N']platinum(II) (2) Geometric parameters (Å, º)

**Table:**

Pt1—N1	2.003 (3)	C2—N2	1.289 (5)
Pt1—N2	2.010 (3)	C2—O2	1.316 (5)
Pt1—As1	2.2672 (8)	N2—H2	0.854 (19)
Pt1—Cl1	2.3387 (11)	C4—C3	1.514 (6)
As1—O6	1.724 (3)	C4—H4B	0.96
As1—O4	1.747 (3)	C4—H4A	0.96
As1—O2	1.946 (3)	C4—H4C	0.96
As1—O1	1.979 (3)	C1—N1	1.303 (5)
O4—H4	0.82	C1—C3	1.500 (5)
O1—C1	1.303 (4)	C3—H3B	0.97
O6—H6	0.82	C3—H3A	0.97
C5—C2	1.498 (5)	N1—H1	0.88 (5)
C5—C6	1.527 (6)	C6—H6B	0.96
C5—H5B	0.97	C6—H6C	0.96
C5—H5A	0.97	C6—H6A	0.96
			
N1—Pt1—N2	173.20 (14)	C2—N2—Pt1	121.9 (3)
N1—Pt1—As1	87.25 (10)	C2—N2—H2	117 (4)
N2—Pt1—As1	86.05 (10)	Pt1—N2—H2	121 (4)
N1—Pt1—Cl1	94.16 (11)	C3—C4—H4B	109.5
N2—Pt1—Cl1	92.53 (10)	C3—C4—H4A	109.5
As1—Pt1—Cl1	178.51 (3)	H4B—C4—H4A	109.5
O6—As1—O4	105.44 (14)	C3—C4—H4C	109.5
O6—As1—O2	86.12 (13)	H4B—C4—H4C	109.5
O4—As1—O2	86.48 (13)	H4A—C4—H4C	109.5
O6—As1—O1	89.28 (12)	C2—O2—As1	116.2 (2)
O4—As1—O1	89.27 (13)	O1—C1—N1	122.0 (4)
O2—As1—O1	172.68 (11)	O1—C1—C3	115.7 (4)
O6—As1—Pt1	130.05 (11)	N1—C1—C3	122.2 (4)
O4—As1—Pt1	124.46 (9)	C1—C3—C4	111.8 (4)
O2—As1—Pt1	94.20 (9)	C1—C3—H3B	109.3
O1—As1—Pt1	93.12 (8)	C4—C3—H3B	109.3
As1—O4—H4	109.5	C1—C3—H3A	109.3
C1—O1—As1	116.5 (2)	C4—C3—H3A	109.3
As1—O6—H6	109.5	H3B—C3—H3A	107.9
C2—C5—C6	115.1 (3)	C1—N1—Pt1	121.1 (3)
C2—C5—H5B	108.5	C1—N1—H1	116 (4)
C6—C5—H5B	108.5	Pt1—N1—H1	123 (4)
C2—C5—H5A	108.5	C5—C6—H6B	109.5
C6—C5—H5A	108.5	C5—C6—H6C	109.5
H5B—C5—H5A	107.5	H6B—C6—H6C	109.5
N2—C2—O2	121.5 (3)	C5—C6—H6A	109.5
N2—C2—C5	124.4 (4)	H6B—C6—H6A	109.5
O2—C2—C5	114.1 (3)	H6C—C6—H6A	109.5
			
C6—C5—C2—N2	−21.3 (6)	As1—O1—C1—N1	−1.4 (5)
C6—C5—C2—O2	159.6 (3)	As1—O1—C1—C3	178.6 (3)
O2—C2—N2—Pt1	−1.3 (5)	O1—C1—C3—C4	63.1 (5)
C5—C2—N2—Pt1	179.8 (3)	N1—C1—C3—C4	−116.9 (4)
N2—C2—O2—As1	3.5 (5)	O1—C1—N1—Pt1	2.7 (5)
C5—C2—O2—As1	−177.5 (2)	C3—C1—N1—Pt1	−177.4 (3)

### Chlorido[dihydroxybis(1-iminopropoxy)arsanido-κN,As,N']platinum(II) (2) Hydrogen-bond geometry (Å, º)

**Table:**

*D*—H···*A*	*D*—H	H···*A*	*D*···*A*	*D*—H···*A*
N1—H1···O6^i^	0.88 (5)	2.19 (5)	3.041 (4)	163 (5)
N2—H2···Cl1^ii^	0.854 (19)	2.71 (4)	3.408 (4)	140 (4)
O4—H4···Cl1^iii^	0.82	2.28	3.071 (3)	161
O6—H6···O1	0.82	2.34	2.608 (4)	100
O6—H6···O4^iv^	0.82	1.92	2.712 (4)	162
C6—H6*A*···Cl1^ii^	0.96	2.82	3.735 (5)	159

Symmetry codes: (i) −*x*+1, *y*−1/2, −*z*+1/2; (ii) −*x*, −*y*, −*z*; (iii) −*x*+1, −*y*, −*z*; (iv) *x*, −*y*+1/2, *z*+1/2.
